# Reliability and validity of the ultrasound technique to measure the rectus femoris muscle diameter in older CAD-patients

**DOI:** 10.1186/1471-2342-12-7

**Published:** 2012-04-02

**Authors:** Tom Thomaes, Martine Thomis, Steven Onkelinx, Walter Coudyzer, Véronique Cornelissen, Luc Vanhees

**Affiliations:** 1 Cardiovascular Rehabilitation Unit, Department of Rehabilitation Sciences, Katholieke Universiteit Leuven, Tervuursevest 101, 3001 Heverlee, Belgium; 2 Exercise and Health Research Group, Department of Kinesiology, Katholieke Universiteit Leuven, Tervuursevest 101, 3001 Heverlee, Belgium; 3 Radiology, University Hospital Leuven, Herestraat 49, 3000 Leuven, Belgium

**Keywords:** Cardiac Rehabilitation, Coronary Artery Disease, Ultrasound Imaging, CT scan

## Abstract

**Background:**

The increasing age of coronary artery disease (CAD) patients and the occurrence of sarcopenia in the elderly population accompanied by 'fear of moving' and hospitalization in these patients often results in a substantial loss of skeletal muscle mass and muscle strength. Cardiac rehabilitation can improve exercise tolerance and muscle strength in CAD patients but less data describe eventual morphological muscular changes possibly by more difficult access to imaging techniques. Therefore the aim of this study is to assess and quantify the reliability and validity of an easy applicable method, the ultrasound (US) technique, to measure the diameter of rectus femoris muscle in comparison to the muscle dimensions measured with CT scans.

**Methods:**

45 older CAD patients without cardiac event during the last 9 months were included in this study. 25 patients were tested twice with ultrasound with a two day interval to assess test-retest reliability and 20 patients were tested twice (once with US and once with CT) on the same day to assess the validity of the US technique compared to CT as the gold standard. Isometric and isokinetic muscle testing was performed to test potential zero-order correlations between muscle diameter, muscle volume and muscle force.

**Results:**

An intraclass correlation coefficient (ICC) of 0.97 ((95%CL: 0.92 - 0.99) was found for the test-retest reliability of US and the ICC computed between US and CT was 0.92 (95%CL: 0.81 - 0.97). The absolute difference between both techniques was 0.01 ± 0.12 cm (p = 0.66) resulting in a typical percentage error of 4.4%. Significant zero-order correlations were found between local muscle volume and muscle diameter assessed with CT (r = 0.67, p = 0.001) and assessed with US (r = 0.49, p < 0.05). Muscle strength parameters were also significantly correlated with muscle diameter assessed with both techniques (range r = 0.45-r = 0.61, p < 0.05).

**Conclusions:**

Ultrasound imaging can be used as a valid and reliable measurement tool to assess the rectus femoris muscle diameter in older CAD patients.

## Background

According to the World Health Association, coronary heart disease (CHD) is the leading cause of death worldwide with age as the most powerful independent risk factor [1]. Ageing is characterized by a decline in functionality due to progressive loss of muscle tissue coupled with a decrease in strength and force output. Low skeletal muscle strength has been shown to be an important predictor of all-cause mortality in healthy as well as diseased individuals [2-4]. The increasing age of coronary artery disease (CAD) patients and the occurrence of sarcopenia in the elderly population accompanied by 'fear of moving' and hospitalization in these patients often results in a substantial loss of skeletal muscle mass and muscle strength.

That is, compared to healthy subjects, CAD patients have an impaired peak VO_2 _and show accompanying increased muscle fatigability [5]. Previous studies have demonstrated that cardiac rehabilitation improves exercise tolerance and muscle strength in patients with myocardial infarction and in patients after cardiac surgery. In addition, Sumide et al. [6] reported that the improvement in exercise tolerance was significantly correlated with the changes in lower limb leg strength in post-cardiac valve surgery patients (r = 0.51, P < 0.01). A positive and significant correlation between the change in peak VO_2 _and the change in peak torque of knee extension (r = 0.50, P < 0.005) was also observed in the acute phase after a myocardial infarction (MI) in patients with a lower limb muscle volume of less than 22 kg at baseline [7].

Repeated ionisation radiation exposure and high costs, accessibility and long scanning times when using CT or MRI, limits the use of both techniques to measure muscle cross sectional area (CSA) and muscle diameter on a broad scale in the clinical and research setting. By contrast ultrasound systems (US) are more easily available and may offer a useful alternative. In asthmatic (mean age 56 ± 8) and chronic obstructive pulmonary disease (COPD) (mean age 67 ± 9) patients it was shown that US can be used as a valid and reliable alternative to CT for measuring mm. rectus femoris (RF) CSA [8,9]. To the best of our knowledge, the validity and reliability of the US technique to measure muscle diameter has not been investigated in an elderly CAD population. Therefore, the aim of this study is to assess and quantify the reliability and validity of the US technique to measure the diameter of RF compared to the muscle dimensions measured with CT scans. In addition, muscle testing was performed to test potential zero-order correlations between muscle diameter, muscle volume and muscle force and to investigate whether correlations found with CT are similar to those with US. Peripheral skeletal muscle strength of the lower limb may be assessed by isokinetic dynamometry and provides a reliable and safe assessment of dynamic muscle function [5,6].

## Methods

### Study sample

Forty five CAD patients (age: 68.4 ± 6.2 years; BMI: 26.6 ± 2.9 kg/m^2^; mean ± SD) without cardiovascular incident during the last year, participating in sporting activities of a maintenance program for patients with cardiovascular disease, volunteered for this study. The first 20 patients (hence forward called 'group 1') (age: 68.3 ± 7.3 years; BMI: 26.8 ± 2.8 kg/m^2^) were measured twice on the same day, once with US and once with CT-scan to investigate the validity of US vs. CT. The following 25 patients (hence forward called 'group 2') (age: 68.6 ± 4.6 years; BMI: 26.3 ± 3.0 kg/m^2^) were measured twice with US with a two day interval to assess the test-retest reliability of this measurement. The study was approved by the Biomedical Ethical Committee of the KU Leuven and written informed consent was obtained from all participants after full explanation of the aims and procedures.

### Measurements

#### Rectus femoris ultrasound

**Figure 1:**
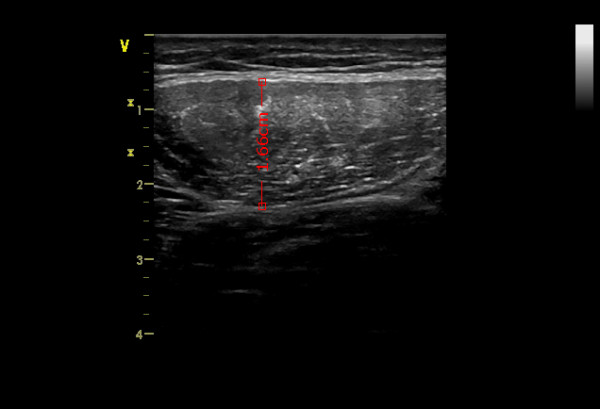
**Image of the rectus femoris with indication of the diameter, obtained with ultrasound imaging**.

All measurements were performed by a single experienced investigator (T.T). Rectus femoris diameter was measured by B-mode ultrasonography, wall tracking ultrasound system (Siemens Vivid 07 GE) with a 12 MHz linear array transducer (12 L transducer GE). The transducer was placed perpendicular to the long axis of the thigh with excessive use of contact gel and minimal pressure to avoid compression of the muscle [8,9]. The diameter of the RF was measured at the half point of the length between epicondylus lateralis and trochanter major of the femur. Measurements were taken on the patient's right leg with the patient lying in a supine position with both knees extended but relaxed and toes pointing the ceiling. A set of five consecutive pictures was taken and further analyzed offline. The vertical diameter of the RF muscle was measured on the inner edge of the muscle on the five pictures (Figure 1). The average of the five pictures was used as the RF diameter and further analyzed. Datasets from both US measurements were analyzed blind and at random.

#### Rectus femoris CT-scan

CT scans were performed using a Siemens Sensation 16^®^. Similarly, measurements of the RF were taken on the right leg at the half point of the length between epicondylus lateralis and trochanter major of the femur with the patient lying in a supine position with both knees extended but relaxed and toes pointing the ceiling. Half way point of the femur was determined using a scout view longitudinal scan of the femur with minimal radiation dose (< 0.05 milliSievert). Five adjacent slices of 0.5 cm thickness were taken (one at mid-point, two directly above and two directly below the midpoint). Additionally femur length, local muscle volume and fat volume in a slice of 2.5 cm (sum of 5 slices) at the middle of the right upper leg were determined with CT (radiation dose < 0.05 milliSievert). Local muscle mass was defined as 0 to 100 Hounsfield Units found in the total leg CSA subtracted with the same densities found in bone marrow. Local fat mass was defined as 0 to -190 Hounsfield Units subtracted with the same densities found in bone marrow. All CT measurements were executed by the same experienced researcher (W.C)

#### Quadriceps strength and anthropometric characteristics

Muscle strength testing was only performed in group 1. After a warming up period of five minutes on a cycle ergometer, maximal voluntary muscle strength of the hamstrings and quadriceps muscles was tested on a BIODEX System 3 Pro (Biodex Medical Systems, 20 Ramsay Road, Shirley, New York, USA). Isometric strength of the quadriceps was measured at 60° (fully extended leg is zero°). Four attempts were given with 30 s interval. The highest peak torque was withheld as the maximal voluntary quadriceps strength. Isokinetic measurements of the quadriceps were measured at 60°/s and 180°/s. Patients performed four consecutive attempts at every speed. Resting interval between both measurements was one minute. Peak torque during both speeds was withheld for further analysis. Vocal encouragement was given by the investigator during the tests.

Finally, height, weight, skinfolds (Harpenden-caliper) and circumference of the mid-thigh and body fat percentage (Omron BF 300; OMRON, Matoukasa Co. Ltd, Japan) were assessed in this group to examine potential associations between anthropometric characteristics and muscle strength, RF diameter, local muscle and fat volume.

#### Statistical analyses

Data were analyzed using SAS statistical software version 9.2 for Windows (SAS Institute Inc, Cary, NC, USA). Data were reported as means ± standard deviation (SD) for anthropometric measurements, RF diameter and muscle strength measurements. The differences between both techniques were reported as means ± SD. The intraclass correlation coefficient (ICC_3,1_) [10] values were computed to assess test-retest reliability of the US technique and the validity of US compared with CT-scan measurements. Additionally a Bland-Altman procedure was used to plot the difference between both techniques compared to the average for all participants and the data was checked for homoscedasticity by means of the correlation between the difference and average scores. Typical error of measurement (TEM) was calculated as the SD of the difference divided by the square root of 2. Zero order correlations (Pearson r) were calculated between anthropometric characteristics and muscle strength. The level of statistical significance was set at p < 0.05.

## Results

A general overview of the descriptive characteristics of all included participants is shown in Table 1.

**Table 1:** Total group patient characteristics

	Mean ± SD or Number (%)
Gender (M/F)	44/1
Age (years)	68.4 ± 6.2
Height (cm)	171.7 ± 5.4
Weight (kg)	78.7 ± 11.3
BMI (kg/m^2^)	26.6 ± 2.9
Time since last cardiac event (years)	6.0 ± 4.1
Past intervention	22 (49)
CABG (N patients)	
PCI (N patients)	22 (49)
Angina Pectoris (N patients)	1 (2)

BMI: Body mass index; CABG: Coronary artery bypass grafting;

PCI: Percutaneous coronary intervention

### Ultrasound versus CT-measured rectus femoris diameter - validity

**Figure 2:**
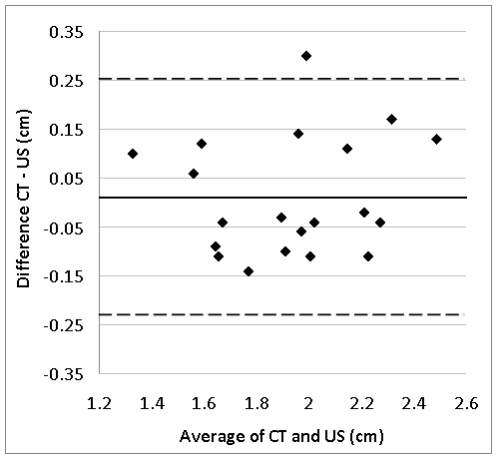
**Bland-Altman plot for the difference between CT scan and Ultrasound for the rectus femoris diameter**. CT: CT-Scan measurement, US: Ultrasound measurement of Rectus femoris. Full 'bold' line: average difference between CT and US. Broken line: limits of agreement.

Baseline characteristics of group 1 are shown in Table 2. Diameter of the RF was 1.937 ± 0.31 cm with CT-scan and 1.925 ± 0.29 cm with US. The average difference (± SD) was non-significant (0.01 ± 0.12 cm, p = 0.66) resulting in a TEM of 0.08 cm or typical percentage error of 4.4%. The ICC between US and CT was 0.92 (95%CL: 0.81 - 0.97). The Bland-Altman plot presenting differences between both measurement procedures against average RF diameter is given in Figure 2. The limits of agreement (LOA) are (0.01 ± 0.24 cm). Only one score is out of the range of LOA. The correlation between the difference and average scores was -0.07 (p = 0.77) indicating homoscedasticity.

**Table 2:** Rectus femoris diameter and patient characteristics in group 1 (N = 20)

	Mean	Std Dev
Height (cm)	172.2	4.5
Weight (kg)	78.1	11.1
Circumference thigh (cm)	50.5	3.4
Skinfold thigh (cm)	1.26	0.47
Body fat percentage (%)	29.0	4.1
Rectus Femoris Diameter with US (cm)	1.925	0.29
CT measurements		
Rectus Femoris Diameter (cm)	1.937	0.31
Femur Length (cm)	46.4	2.1
MuscleVolume (cm^3^)	308	43.6
Fat Volume (cm^3^)	116	40.8
Muscle strength		
Isometric extension 60° (Nm)	181	26
Isokinetic flexion 60°/s (Nm)	77.4	16.4
Isokinetic flexion 180°/s (Nm)	64.7	14.1
Isokinetic extension 60°/s (Nm)	129	21
Isokinetic extension 180°/s (Nm)	85.2	15

US: Ultrasound; CT: Computed tomography

Results of the zero-order correlations between patient characteristics and muscle strength parameters are shown in Table 3. Highest correlations were found between muscle volume of the thigh and diameter of RF measured by CT with all muscle strength parameters. Correlations of diameter RF, measured by US, with strength parameters were lower than those of CT with strength. Muscle volume of the mid-thigh region correlated significantly with RF diameter measured with CT (r = 0.67, p = 0.001) and measured with US (r = 0.49, p < 0.05).

**Table 3:** Zero-order correlations between rectus femoris diameter, anthropometric characteristics and muscle strength parameters in group 1

	Isometric extension (Nm)	Isokinetic flexion 60°/s (Nm)	Isokinetic flexion 180°/s (Nm)	Isokinetic extension 60°/s (Nm)	Isokinetic extension 180°/s (Nm)
Age (years)	-0.18	-0.47*	-0.38	-0.18	-0.29
Weight (kg)	0.52*	0.31	0.38	0.42	0.42
Height (cm)	0.79**	0.12	0.30	0.50*	0.58**
Femur length (cm)	0.65**	0.06	0.05	0.51*	0.42
Fat volume thigh (cm^3^)	-0.05	-0.30	-0.15	-0.11	-0.22
Body fat percentage (%)	-0.16	-0.28	-0.14	-0.12	-0.28
Skinfold thigh (cm)	-0.03	-0.13	-0.26	-0.08	-0.25
Circumference thigh (cm)	0.50*	0.39	0.45*	0.49*	0.46*
Muscle volume thigh (cm^3^)	0.62**	0.75***	0.61**	0.68***	0.69***
RF diameter CT (cm)	0.69***	0.66**	0.67**	0.63**	0.74***
RF diameter US (cm)	0.52*	0.54*	0.61**	0.45*	0.59**

*p < 0.05, **p < 0.01,***p < 0.001

### US RF diameter test-retest reliability

**Figure 3:**
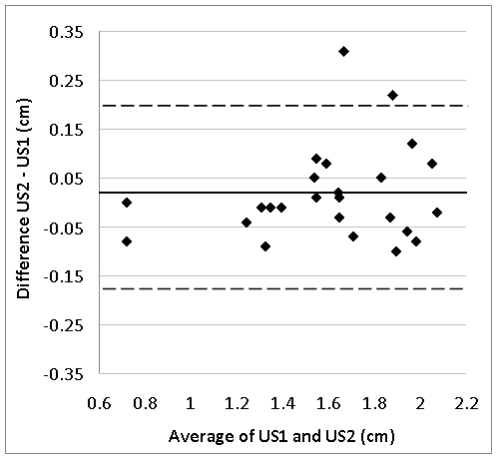
**Bland-Altman plot for the difference between both US measurements for the RF diameter**. US1: Ultrasound measurement 1, US2: Ultrasound measurement 2. Full 'bold' line: average difference between Ultrasound measurement 1 and 2. Broken line: limits of agreement.

Table 4 shows the results of the two US measurements of the RF of 25 patients on two separate days. The difference between both measurements was non-significant (0.02 ± 0.10 cm, p = 0.4) with a TEM of 0.07 or a typical percentage error of 4.2%. The ICC was 0.97 (95%CL: 0.92 - 0.99) between the two measurements. The Bland-Altman plot presenting differences between both measurements against the average for both measurements is given in Figure 3. Two scores fall outside the boundaries of LOA. Minimal detectable difference (MDD) for this group of patients (N = 25) was 0.24 cm.

**Table 4:** Test - retest reliability of the ultrasound measurement in group 2

Patient	Measurement 1	Measurement 2	Difference
1	1.97	1.91	-0.06
2	1.4	1.39	-0.01
3	1.55	1.63	0.08
4	1.26	1.22	-0.04
5	1.77	1.99	0.22
6	2.01	2.09	0.08
7	2.02	1.94	-0.08
8	1.51	1.56	0.05
9	1.88	1.85	-0.03
10	1.51	1.82	0.31
11	1.31	1.3	-0.01
12	1.5	1.59	0.09
13	1.54	1.55	0.01
14	1.63	1.65	0.02
15	1.37	1.28	-0.09
16	1.64	1.65	0.01
17	1.9	2.02	0.12
18	1.74	1.67	-0.07
19	1.8	1.85	0.05
20	2.08	2.06	-0.02
21	1.94	1.84	-0.1
22	1.35	1.34	-0.01
23	1.66	1.63	-0.03
24	0.76	0.68	-0.08
25	0.72	0.72	0

Average	1.593	1.609	0.02 ± 0.10^NS^

^NS^: not significant

## Discussion

This study shows that US is a valid and reliable tool to measure the diameter of the RF in stable, elderly CAD patients. It further shows that the diameter of RF, measured with US, is significantly correlated with different muscle strength parameters.

We found a high ICC for both test-retest of the measurement of the RF with US (0.97) as for the comparison of RF measurements with US and CT (0.92) in these older cardiac patients. This is in line with the study of Seymour et al. [9] who reported in COPD patients an ICC of 0.97 for test-retest reliability of US measurement of rectus femoris CSA and of 0.88 for validity of the US measurement of rectus femoris CSA compared with CT. Also Bemben et al. [11] and Kanehisa et al. [12] reported an ICC of 0.72 and 0.99 respectively for test-retest reliability of the CSA measurements using B-mode US technique in various age groups. Bemben et al. tested US and MRI reliability for muscle CSA of the RF at 15 cm above the patella and found no significant differences between both techniques in young subjects (age: 26 years). Similar results were found for the reliability of the US measurement of the vastus lateralis muscle (ICC between 0.997 and 0.999) and for the validity compared with MRI scans (ICC between 0.998 and 0.999) [13].

All patients included in this study were CAD patients without cardiac event during the last 9 months, who participated in at least one session of exercise training per week under supervision of a physiotherapist, and could therefore be considered to be still fairly active elderly.

The observed diameter of RF in our sample of cardiac patients is comparable to earlier findings. That is, Delaney et al. [14] showed a RF depth of 2.3 cm in resting position in healthy young males (mean age 24.6 years). Arts et al. [15] found a quadriceps diameter (thickness of rectus femoris + vastus intermedius) in males of 4.16 ± 1.02 cm (age range 17-90 years) whereas Nogueira et al. [16] found a RF diameter of 1.86 cm in 20 older men (age 69-76 years).

In addition we investigated the relation between muscle diameter, muscle force and muscle volume in this cohort. We found strong correlations (0.61-0.75) between muscle volume and diameter assessed with CT and all the muscle strength parameters. Muscle diameter assessed with US also significantly correlated (0.45-0.61) with all strength measures, although the correlations where somewhat less as compared to CT. Muscle volume of the mid-thigh region correlated significantly (r = 0.67, p = 0.001) with RF diameter (CT-technique), which was comparable to the results reported by Seymour et al. [9] in a healthy control group. RF diameter measured with US also significantly correlated with muscle volume (r = 0.49, p < 0.05). The reason for the less strong correlations when RF diameter was assessed by the US technique could be due to the higher variability of consecutive measurements in US (compression of the muscle tissue, deviation from perpendicular viewing). The more accurate determination of the middle of the femur using CT (visualization of the bone structure by means of the scout view) compared with surface determination of bony landmarks in US could also be an important factor.

Earlier, the US technique has shown to be a valid and reliable alternative to CT or MRI in studies comparing muscle RF CSA of COPD patients with healthy controls [9] and in a study to determine the effects of resistance training on muscle thickness or muscle volume in older men [16]. In the latter study, an increase of 0.21 cm was found in the high velocity power training group, which is comparable with the MDD of 0.24 cm we found for test-retest reliability.

## Conclusions

Nowadays, guidelines recommend the inclusion of resistance exercises in rehabilitation programs of cardiac patients. The observed validity and reliability make the use of an ultrasound device in cardiac rehabilitation an interesting tool to measure (changes in) muscle mass following exercise in all phases of cardiac rehabilitation.

## Competing interests

The authors declare that they have no competing interests.

## Authors' contributions

TT analyzed and interpreted the data, performed statistical analysis, and drafted the manuscript. MT performed statistical analyses, assisted with interpretation of the data. SO assisted with the collection and analyses of the data. WC acquired and interpreted the CT scan. VC assisted with interpretation of the data. LV conceived and designed the research. All authors read, approved and contributed to the manuscript.

## Pre-publication history

The pre-publication history for this paper can be accessed here:

http://www.biomedcentral.com/1471-2342/12/7/prepub
